# Function and Regulation of *Vibrio campbellii* Proteorhodopsin: Acquired Phototrophy in a Classical Organoheterotroph

**DOI:** 10.1371/journal.pone.0038749

**Published:** 2012-06-07

**Authors:** Zheng Wang, Thomas J. O'Shaughnessy, Carissa M. Soto, Amir M. Rahbar, Kelly L. Robertson, Nikolai Lebedev, Gary J. Vora

**Affiliations:** 1 Center for Bio/Molecular Science and Engineering, Naval Research Laboratory, Washington, D.C., United States of America; 2 National Cancer Institute, Bethesda, Maryland, United States of America; National Institute on Drug Abuse, United States of America

## Abstract

Proteorhodopsins (PRs) are retinal-binding photoproteins that mediate light-driven proton translocation across prokaryotic cell membranes. Despite their abundance, wide distribution and contribution to the bioenergy budget of the marine photic zone, an understanding of PR function and physiological significance *in situ* has been hampered as the vast majority of PRs studied to date are from unculturable bacteria or culturable species that lack the tools for genetic manipulation. In this study, we describe the presence and function of a horizontally acquired PR and retinal biosynthesis gene cluster in the culturable and genetically tractable bioluminescent marine bacterium *Vibrio campbellii*. Pigmentation analysis, absorption spectroscopy and photoinduction assays using a heterologous over-expression system established the *V. campbellii* PR as a functional green light absorbing proton pump. *In situ* analyses comparing PR expression and function in wild type (WT) *V. campbellii* with an isogenic Δ*pR* deletion mutant revealed a marked absence of PR membrane localization, pigmentation and light-induced proton pumping in the Δ*pR* mutant. Comparative photoinduction assays demonstrated the distinct upregulation of *pR* expression in the presence of light and PR-mediated photophosphorylation in WT cells that resulted in the enhancement of cellular survival during respiratory stress. In addition, we demonstrate that the master regulator of adaptive stress response and stationary phase, RpoS1, positively regulates *pR* expression and PR holoprotein pigmentation. Taken together, the results demonstrate facultative phototrophy in a classical marine organoheterotrophic *Vibrio* species and provide a salient example of how this organism has exploited lateral gene transfer to further its adaptation to the photic zone.

## Introduction

Bacterial proteorhodopsins (PRs) are membrane embedded, retinal-binding ion transporters that can create a proton electrochemical potential for ATP production in response to specific wavelengths of light [1], [2]. Although bacterial PRs were first discovered in an uncultivated member of the gammaproteobacterial "SAR86"; group [1], subsequent marine metagenomic and completed genome sequence analyses have revealed PRs in an assortment of uncultivated alphaproteobacteria [3] and euryarchaeotes [4], and cuturable alphã, betã, gammaproteobacteria [5], [6], [7], [8], Bacteroidetes [6], [9], [10], [11] and marine dinoflagellates [12]. As the vast majority of PRs discovered have been attributed to unculturable and/or genetically intractable organisms, most functional characterizations have required the use of *E. coli* as a heterologous experimentation system. Such studies have convincingly demonstrated PR and retinal-mediated pigmentation via genetically linked retinal biosynthetic pathways [13], [14], PR proton pumping [1], [14], photocycling rates characteristic of transporter rhodopsins [1], PR-mediated photophosphorylation [14] and the ability of PR to sustain cellular proton motive force during periods of respiratory distress [15]. PR function has also been interrogated *in situ* in a few culturable marine bacterial species that lack the tools for genetic manipulation and these studies have demonstrated the expression and membrane localization of PR in the SAR 11 clade member *Pelagibacter ubique* (HTCC1062) [5], PR photocycling in HTCC1062 and SAR92 clade member HTCC2207 [5], [7] and increased light-dependent *pR* expression and cell growth in *Dokdonia sp.* MED134 cultures [9], [16] and SAR11 and *Flavobacteria* containing coastal water microcosms [17]. While informative, the current inability to genetically manipulate these species has limited the power of these studies to decipher the direct role of PR in marine bacterial physiology.

Recently, the culturable and genetically tractable marine bacterium *Vibrio* sp. AND4 was also shown to harbor a functional *pR* and retinal biosynthesis gene cluster and in this case the ability to generate an in-frame *pR* deletion mutant allowed for the direct testing of a physiological role for PR *in situ* [8]. While AND4 growth experiments in rich medium in the presence and absence of light showed no difference in cellular yield, the illumination of starved cells in seawater led to the increased survival of *Vibrio* sp. AND4 and this response was shown to be specifically mediated by the *pR* gene product [8]. Furthermore, starved but illuminated *Vibrio* sp. AND4 cells also demonstrated a more rapid growth recovery in improved conditions than cells that were not exposed to light [8]. Importantly, the observed effects in the absence of PR (Δ*prd*) were found to be rescued by *prd* complementation *in trans*. Thus the experimental combination of deletion mutagenesis and starvation/growth recovery cultures provided strong evidence of phototrophy, specifically the ability to use light to survive starvation, in a member of the organoheterotrophic *Vibrionaceae*.

In this study, we reveal the presence, function and regulation of a horizontally acquired green light absorbing PR proton pump from a closely related sister species to *Vibrio* sp. AND4, the bioluminescent marine bacterium *Vibrio campbellii* strain BAA-1116 [18]. In addition to confirming several basic aspects of PR biology *in situ* (e.g. *pR* transcription and translation, membrane pigmentation, photoinduced proton pumping, spectral tuning, PR-mediated ATP production and enhancement of cell survivability during respiratory challenge), we also identify the first regulator of *pR* expression: the alternative sigma factor and master regulator of the adaptive stress response and stationary phase, RpoS1, positively regulates *pR* expression and PR holoprotein pigmentation.

## Results

### Genetic locus, sequence and phylogeny

The genome of *V. campbellii* strain BAA-1116 revealed an ORF on chromosome I that was identified as a PR encoding gene (chr1-orf00617, GenBank accession number FJ985782). An examination of the neighboring ORFs revealed that the *pR* gene is immediately flanked by a series of transposases and five genes that putatively encode the enzymes geranylgeranyl diphosphate synthase (CrtE), phytoene desaturase (CrtI), phytoene synthase (CrtB), lycopene cyclase (CrtY) and 15,15′-ß-carotene dioxygenase (Blh) necessary for ß-carotene and retinal biosynthesis from farnesyl diphosphate and isopentenyl diphosphate [13], [19] (Figure S1a). Four highly conserved sequence features (seven transmembrane alpha helices, signal peptide [20], conserved AA residues [21] and a leucine residue corresponding to PR position 105 [22]) strongly suggested that orf00617 likely encoded a PR proton pump that is spectrally tuned to green light (Figure S1b). Phylogenetic analysis of the *V. campbellii* PR AA sequence demonstrated that it formed a distinct cluster with other green light absorbing PR orthologs and was most similar (87% identity) to the PR found in the genome sequenced *Vibrio* sp. AND4 (Figure S1c), a finding that confirms that of a previous study [8]. Interestingly, two other sequenced members of the family *Vibrionaceae*, *V. angustum* S14 and *Photobacterium sp.* SKA34, also harbor putative PRs; however, these PRs are predicted to be blue light absorbing and did not cluster with the putative green light absorbing *V. campbellii* PR. This was not unexpected as it has previously been demonstrated that *pR* gene phylogeny does not parallel organismal evolutionary relatedness [4], [23], [24] and was consistent with the assertion that the *V. campbellii* PR and retinal biosynthesis genes have been acquired via lateral transfer [23], [24].

### Heterologous expression and function of *V. campbellii* PR in *E. coli*

A heterologous *V. campbellii* PR over-expression system was established in *E. coli* to verify the sequence-predicted function of *V. campbellii* PR. The induction of two *pR*-containing plasmid constructs (PR_Met1_ and PR_Leu20_) resulted in the characteristic pink/orange membrane pigmentation indicative of the retinal bound PR holoprotein (Figure S2a). Western blot analyses of these same cell pellets using a His_6_-tag monoclonal antibody and anti-PR peptide-specific antibody confirmed the expression of PR and revealed the presence of a signal peptide in *V. campbellii* PR (Figure S2b). When *E. coli* PR_Met1_ and BL21 (control) cell suspensions containing exogenous all-*trans* retinal were exposed to white light, a light-dependent acidification of the medium was seen only in the *E. coli* PR_Met1_ cultures (Figure S2c) and the absorption spectrum of cell membranes from these suspensions demonstrated that PR_Met1_ had an absorption maximum of 523 nm (Figure S2d). Finally, we demonstrated spectrally-tuned proton pumping by sequentially exposing *E. coli* PR_Met1_ cell suspensions to 10.5 mW of red light (670±20 nm) and 5.8 mW of green light (530±17.5 nm) (Figure S2e).

### Expression and functional analyses of *V. campbellii* PR *in situ*

**Figure 1:**
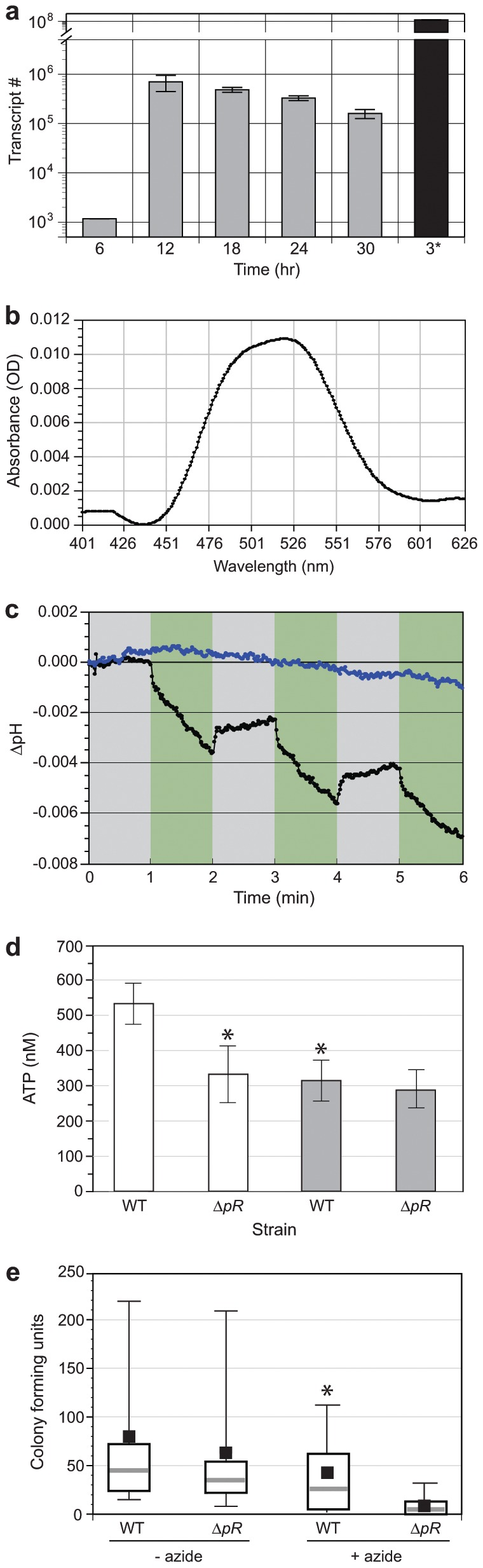
Proteorhodopsin expression and function in *V. campbellii*. (a) Time course analysis of *pR* transcription via reverse-transcription real-time PCR. Gray bars, *V. campbellii* WT; black bar, *E. coli* PR_Met1_ at 3 hr post-induction. (b) Absorption spectrum of pigmented *V. campbellii* WT cells demonstrating an *in situ* λ_max_ of 520 nm. (c) Photoinduced proton pumping by *V. campbellii* WT cell suspensions in the absence of all-*trans* retinal. Changes in pH were monitored in 1 min intervals in the absence (gray regions) or presence of green light (530±15.0 nm, 20.0 mW, green regions). Black line–*V. campbellii* WT; blue line–*V. campbellii* Δ*pR*. (d) PR-mediated photophosphorylation when illuminated with green light (530±15.0 nm, 20.0 mW). White bars–illuminated cells; gray bars – non-illuminated cells. Data shown represent means ± SD of triplicate determinations. *, *P*<0.02 versus WT illuminated cells (Student's *t* test). (e) *V. campbellii* WT and Δ*pR* cell survival post azide-mediated respiratory challenge. Gray lines – median; black boxes – mean; open boxes – top and bottom quartiles; bars – top and bottom deciles. *, *P*<0.01 versus Δ*pR* + azide (two-sided Mann-Whitney *U* test).

Having demonstrated *V. campbellii* PR function in a heterologous over-expression system, we sought to determine whether the *pR* gene was expressed and functional *in situ*. *V. campbellii* WT cells were cultured in colorless autoinducer bioassay (AB) medium in the dark and harvested every 6 hr for quantitative reverse-transcription *pR* PCR (qRT-PCR) analysis. The *pR* expression profile revealed minimal expression in lag phase growth (6 hr) and peak expression during mid-log phase (12 hr) growth followed by a steady decline in transcript number through stationary phase (Figure 1a). A comparison of *V. campbellii* WT mid-log phase *pR* expression with the gratuitously induced *E. coli* PR_Met1_ expression at 3 hr post-induction demonstrated that the heterologous over-expression system generated ∼192× more *pR* mRNA than *V. campbellii*. Thus, these results not only provided evidence of *pR* expression *in situ* but also suggested why *V. campbellii* WT cells never exhibited the intense pink/orange PR holoprotein pigmentation that was seen in the *E. coli* PR_Met1_ over-expression system. However, when cell pellets from WT *V. campbellii* and its isogenic *pR* deletion mutant (Δ*pR*) were harvested from overnight AB medium cultures grown in the absence of exogenous retinal, the WT cells demonstrated a distinct pink tinge that was markedly absent in Δ*pR* cells (data not shown). These results not only suggested that *V. campbellii* PR formed functional holoproteins *in situ* but also provided evidence that the linked retinal biosynthetic pathway is functional as pigmentation was seen in the absence of exogenous retinal [13]. Subsequent mass spectrometry analyses of the WT and Δ*pR* membrane fractions identified a single tryptic PR peptide from WT membranes (DVWVATGETPTVYR, Asp_101_–Arg_114_) that was detected with high confidence (>99%) and notably absent in the Δ*pR* membrane fraction.

The absorption spectrum of the WT cell membranes demonstrated an absorption maximum of 520 nm (Figure 1b). As the absorption maximum seen *in situ* was similar to that seen in *E. coli* PR_Met1_, we similarly tested the ability of *V. campbellii* WT cell suspensions to pump protons when illuminated with green wavelengths of light. The exposure of WT cell suspensions to 20.0 mW of green light (530±15.0 nm) resulted in photoinduced proton pumping and accompanying pH changes in the absence of exogenous all-*trans* retinal (Figure 1c). There was no photoinduced proton pumping seen in Δ*pR* cell suspensions.

The generation of a proton motive force via light-activated PR proton pumping has been shown to be sufficient to drive ATP synthesis in an *E. coli* PR over-expression system [14]. Our demonstration of photoinduced proton pumping in WT cell suspensions led us to test whether we could similarly measure changes in ATP levels in a native PR photosystem. Measurements of cellular ATP levels from WT and Δ*pR* cells that were first simultaneously illuminated for 10 min using 20.0 mW of green light (530±15.0 nm) revealed a significant increase of light-induced ATP levels in the WT cells when compared to illuminated Δ*pR* cells and non-illuminated WT and Δ*pR* cells (Figure 1d). The PR-mediated photophosphorylation resulted in an average 61% increase of cellular ATP levels in WT cells or an increase of 10 fM ATP per cell when compared to illuminated Δ*pR* cells.

The ability to sustain a light-driven chemiosmotic potential that results in photophosphorylation could potentially serve as a competitive advantage when adapting to nutrient poor and light intense environments such as the oligotrophic marine photic zone. To determine whether *V. campbellii* PR could play a role in enhancing viability in nutrient-depleted environments, we tested the ability of illuminated WT and Δ*pR* cells to survive in artificial seawater lacking a carbon source while in the presence or absence of the respiratory chain poison azide. Illuminated WT and Δ*pR* cells in a nutrient-depleted medium in the absence of azide demonstrated similar rates of survival (Figure 1e). However, when simultaneously presented with an azide-mediated respiratory challenge, WT cells demonstrated an enhanced ability to survive the challenge when compared to Δ*pR* cells. These results are consistent with previous findings that have demonstrated a PR-mediated enhancement of cell survivability during respiratory challenge [15] or nutrient-limiting conditions [8], [25] and suggest that *V. campbellii* PR provides a means of generating amounts of ATP that are sufficient to sustain cellular viability during periods of low or inadequate oxidative phosphorylation activity.

### Effect of light on *V. campbellii* growth and *pR* expression

**Figure 2:**
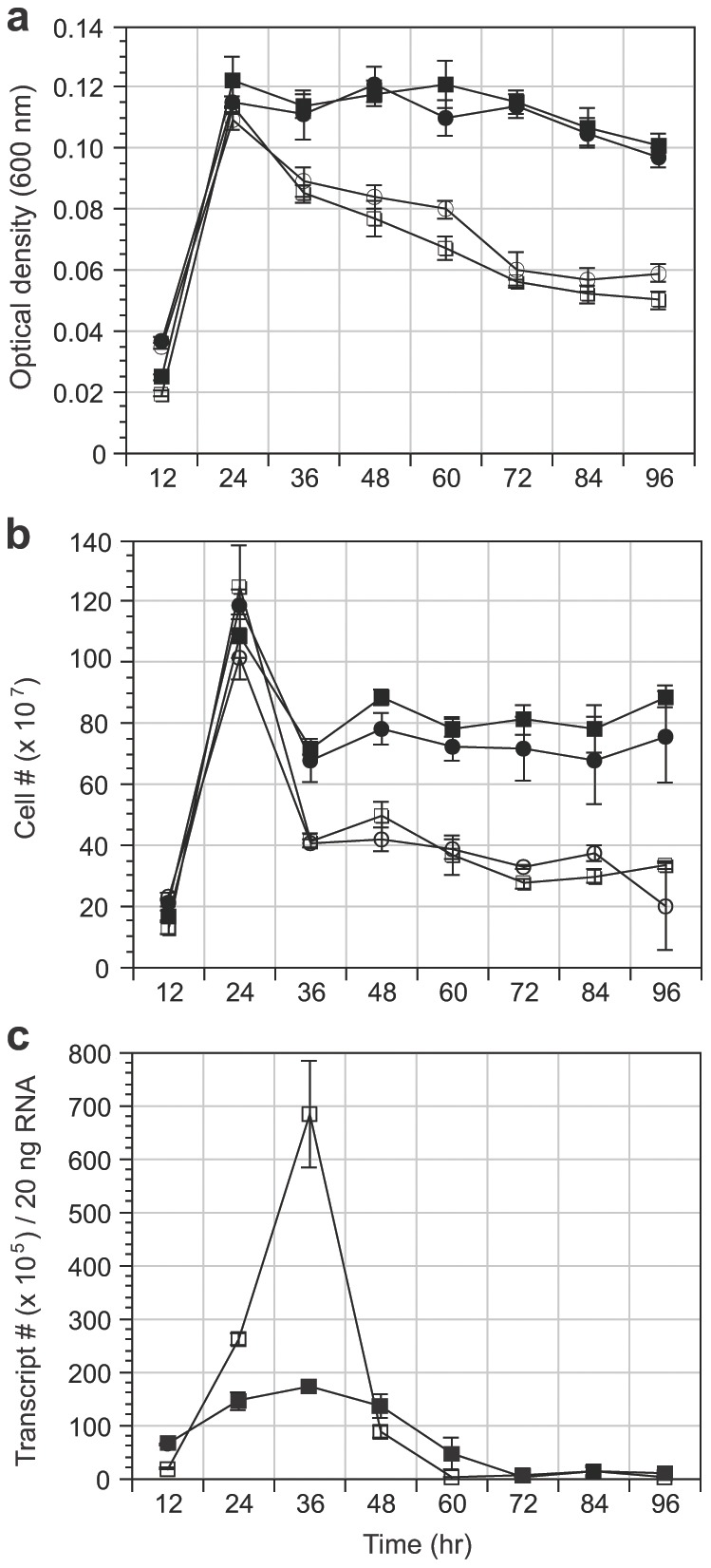
Influence of continuous light or dark on *V. campbellii* WT and Δ*pR* growth in minimal medium and *pR* transcription. White squares–*V. campbellii* WT in continuous light; black squares–*V. campbellii* WT in continuous dark; white circles–*V. campbellii* Δ*pR* in continuous light; black circles–*V. campbellii* Δ*pR* in continuous dark. (a) Optical density measurements (OD_600_) and (b) Flow cytometry-based assessment of bacterial growth in M9 minimal medium cultures. Data shown represent means ± SD of duplicate determinations from two experiments. (c) Time course analysis of *V. campbellii* WT *pR* transcription via reverse-transcription real-time PCR from M9 minimal medium cultures grown in continuous light or dark.

The ability of *V. campbellii* PR to translocate protons, photophosphorylate and enhance survival rates during respiratory distress *in situ* led us to investigate whether light played a role in the growth of *V. campbellii* or in the regulation of the *pR* gene. *V. campbellii* WT and Δ*pR* cultures were grown in a defined minimal medium under continuous illumination (42 µmol photons s^−1^ m^−2^, photosynthetically active radiation) or continuous dark and sampled every 12 hr for optical density measurements, flow cytometry-based cell counts and qRT-PCR. Optical density measurements (Figure 2a) and flow cytometry-based cell counts (Figure 2b) indicated that the cellular yield of each culture peaked at 24 hr. While all four cultures demonstrated a reduction in cell numbers at 36 hr both illuminated cultures showed the greatest reduction in cell numbers (WT-67%, Δ*pR*-60%) (Figure 2b). This trend continued through 96 hr at which time there were 62% fewer cells in the illuminated WT cultures versus the dark WT cultures and 74% fewer cells in the illuminated Δ*pR* cultures versus the dark Δ*pR* cultures. Rather surprisingly, these results indicate that under the experimental conditions used, the effect of continuous light on *V. campbellii* results in mortality and dominates any measurable contribution by PR (if any at all) for the enhancement of cellular yield. A qRT-PCR time course comparison of illuminated and dark WT cultures revealed a 1.8× and 3.9× increase in *pR* transcripts in illuminated cultures at 24 hr and 36 hr, respectively (Figure 2c). This result suggested that *V. campbellii pR* expression is induced by light and is in agreement with previous findings [9], [16], [17]. Interestingly, the number of *pR* transcripts peaked at 36 hr for both illuminated (37.3× compared to the illuminated 12 hr time point) and dark (2.7× compared to the dark 12 hr time point) cultures also suggesting that in addition to light, the stress associated with entry into stationary/starvation phase may also play a role in the regulation of the *pR* gene.

### Regulation of *pR* expression by the alternative sigma factor RpoS1

**Figure 3:**
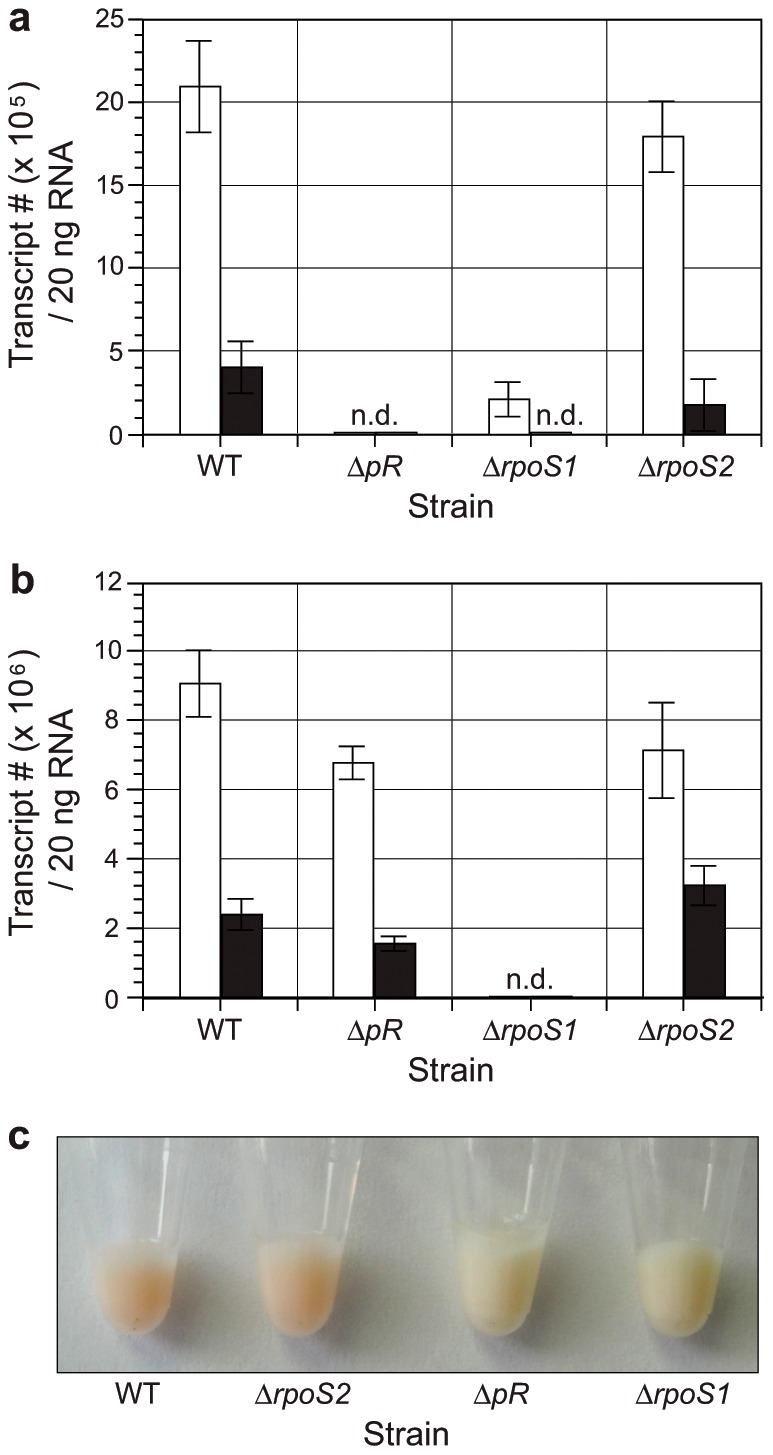
RpoS1 as an activator of *pR* expression. (a) *V. campbellii* WT, Δ*pR*, Δ*rpoS1* and Δ*rpoS2* M9 minimal medium stationary phase cultures (36 hr) that were either continuously illuminated or dark were assessed by reverse-transcription real-time PCR for *pR* expression or (b) *rpoS1* expression. White bars–continuously illuminated cultures; black bars–continuously dark cultures; n.d. – none detected. Data shown represent means ± SD of two technical replicates from two independent experiments. (c) Comparison of PR-mediated pigmentation in *V. campbellii* WT, Δ*pR*, Δ*rpoS1*, and Δ*rpoS2* stationary phase cells grown under continuous illumination.

As the alternative sigma factor RpoS is known in vibrios to be the central regulator of adaptive stress response and stationary/starvation phase persistence in the marine environment, we investigated whether the *V. campbellii* BAA-1116 RpoS ortholog (RpoS1) or the horizontally-acquired RpoS xenolog (RpoS2) were involved in the regulation of the *pR* gene. *V. campbellii* WT and its isogenic deletion mutants Δ*pR*, Δ*rpoS1* and Δ*rpoS2* were cultured in minimal medium under continuous white light illumination (42 µmol photons s^−1^ m^−2^) or continuous dark and sampled at 36 hr for qRT-PCR analyses. As expected, a comparison of *pR* transcript levels in illuminated versus dark cultures revealed that illuminated cells contained significantly more *pR* transcripts then their non-illuminated counterparts (WT, *P*<0.0001; Δ*rpoS1*, *P*<0.005; Δ*rpoS2*, *P*<0.0001; two-tailed Student's *t* test) (Figure 3a). Additional comparisons demonstrated that illuminated WT cells contained 12.0× more *pR* transcripts than illuminated Δ*rpoS1* cells (*P*<0.0001) and dark WT cells also contained significantly more *pR* transcripts than dark Δ*rpoS1* cells (*P*<0.0001). Thus, each comparison demonstrated that *pR* transcription was significantly decreased in the absence of light or RpoS1 and was not detectable when both light and RpoS1 were absent. The absence of the alternative sigma factor RpoS2 had no effect on *pR* transcription. A similar qRT-PCR analysis was performed to compare *rpoS1* transcript levels in illuminated versus dark cultures using all four strains. In each case, the illuminated cells contained 2.1–4.4× more *rpoS1* transcripts than cells grown in the dark (*P*<0.008) (Figure 3b). These observations are consistent with the contention that RpoS1 positively regulates *pR* expression and that light exacerbates the *V. campbellii* stationary phase stress response.

To validate the RpoS1-mediated *pR* expression findings, we performed a pigmentation analysis. When all four strains were grown to stationary phase only the WT and Δ*rpoS2* strains produced pink-tinged cell pellets (Figure 3c). In addition, the supplementation of Δ*rpoS1* cultures with 10 µM all-*trans* retinal did not result in pink-tinged cell pellets suggesting that this lack of pigmentation was not due to the absence of retinal (data not shown). The pigmentation phenotypes observed support the gene expression data and suggested that RpoS1, and not another alternative sigma factor such as RpoS2, positively regulates the *V. campbellii pR* gene.

## Discussion

Members of the genus *Vibrio* are among the best-studied inhabitants of the marine photic zone due to their global distribution, ease of isolation, abundance, genetic tractability and capacity to cause disease in aquatic animals and humans [26]. They have also been described as ‘opportuni-trophs’ as their genomic plasticity has enabled their adaptation to a variety of dynamic environmental niches [27]. In this study, we bolster that assertion by demonstrating that *V. campbellii* strain BAA-1116 has acquired functional *pR* and retinal biosynthesis genes that impart the ability to generate biochemical energy from light energy. In doing so, we find that *V. campbellii* BAA-1116 [like *Vibrio* sp. AND4 (Gomez-Consarnau *et al.*, 2010)] is a mixotrophic bacterium (specifically a photo(organo)heterotroph) [28], that has exploited lateral gene transfer to further its adaptation to the often oligotrophic marine photic zone. This metabolic flexibility, combined with its comparatively rapid division times and ability to withstand long periods of stress in the viable but nonculturable state, may allow *V. campbellii* an enhanced ability to survive in nutrient poor and light intense environments.

The full complement of genetic and proteomic tools available for *V. campbellii* provided us with a rare opportunity to understand PR expression, functionality and regulation *in situ* as opposed to exclusive demonstrations of functionality using heterologous over-expression systems. In many ways our *in situ* results are consistent with previous findings in heterologous over-expression systems that have demonstrated *pR* transcription and translation, membrane pigmentation [1], spectral tuning [22], photoinduced proton pumping [1], PR-mediated ATP production [14], and PR-mediated enhancement of cell survivability during respiratory challenge [15] or nutrient-limiting conditions [8], [25]. However, our finding that continuous illumination actually decreased *V. campbellii* BAA-1116 cellular yields is in contrast to results found in other bacterial systems. For example, in *Vibrio* sp. AND4 illumination of starved cells in seawater led to their increased survival and starved but illuminated cells also demonstrated a more rapid growth recovery in improved conditions than cells that were not exposed to light [8]. Similarly, the exposure to certain photic and nutrient-limiting conditions have resulted in increased cellular yields in some PR-containing systems [9], [16], [17], but not in others [5], [6], [7], [11], [29], [30]. In contrast to these positive or neutral responses to illumination, our results demonstrated a negative response to continuous illumination as both *V. campbellii* WT and Δ*pR* cultures grown in nutrient limited media yielded fewer cells than their non-illuminated counterparts. Although unexpected, we speculate that this decline in cell number may be the result of the prolonged and continuous exposure to light stress which may lead to the accumulation of deleterious effects on cellular components and/or the light stress-mediated induction of lytic phage [31] as the *V. campbellii* BAA-1116 genome is known to harbor four chromosomally integrated phage genomes. This contention is supported by a recently conducted expression profiling study which sought to determine the *V. campbellii* photostimulon. Using the same light intensity that was used in this study (which corresponds to the amount of light found 50–75 m below the sea surface at mid-day on a bright clear day), both constitutively illuminated and cycling 12 h light∶12 h dark cultures demonstrated a significant modulation of genes encoding heat shock proteins, photolyases, DNA repair proteins, stress response sigma factors thus supporting the role of light as a stressor for this organism (manuscript in preparation). Thus the direct or indirect results of light as a stressor may overwhelm any measurable benefit provided by PR under the experimental conditions utilized. The disagreement in this result with those of others may also be due to varying experimental conditions or the specific *V. campbellii* physiological and genomic context (Martinez *et al.*, 2007).

Much like the varying influence of light on the cellular yield of *pR*-containing organisms, light also appears to generate varied responses in *pR* expression. In some bacteria light has been shown to enhance *pR* expression (e.g. *Dokdonia sp.* MED134 [9], [16] and SAR11/*Flavobacteria-*containing coastal water microcosms [17]), while in others (i.e. *D. donghaensis* strain PRO95) *pR* is expressed constitutively and is not affected by light or carbon concentration [11]. In *V. campbellii*, not only did illuminated minimal medium cultures demonstrate peak *pR* expression in stationary phase but dark cultures also demonstrated peak *pR* expression in stationary phase. This finding led us to hypothesize that *V. campbellii pR* transcription may not only be induced by light but also by the cellular stationary/starvation phase stress response – to which light acts as an exacerbating stressor. While the molecular regulators responsible for *pR* expression are currently not known in any other *pR*-containing organism, our use of deletion mutagenesis, qRT-PCR and pigmentation analysis have revealed that RpoS1, the central regulator of the adaptive response to oxidative stress and stationary/starvation phase stress, positively regulates *pR* expression in *V. campbellii*. Light appears to exacerbate the *V. campbellii* stationary phase stress response which results in the increased transcription of *rpoS1* and in turn, *pR*, but it remains to be determined whether RpoS1 is directly or indirectly responsible for *pR* transcription. While currently unknown, efforts are underway to identify the *V. campbellii* RpoS1 promoter consensus sequence via Δ*rpoS1* microarray-based expression profiling and bioinformatic promoter prediction. Furthermore, while the data does suggest that RpoS1 is responsible for the majority of *pR* transcription, the detection of transcripts in illuminated stationary phase cultures in the absence of RpoS1 (Δ*rpoS1*) also suggests that a second mechanism of regulation likely also contributes to *pR* expression.

*V. campbellii* BAA-1116 has acquired a functional *pR* and retinal biosynthesis gene cluster that enables the exploitation of light as a source of energy in the photic zone. However, expansion into this niche inherently requires the ability to deal with light-mediated stress as solar radiation on surface waters can cause direct (UV radiation-based DNA mutagenesis) and indirect (formation of peroxides and oxygen radicals) photochemical damage to cellular components. *V. campbellii* appears to have coupled *pR*-mediated resource utilization with stress management by integrating the horizontally acquired *pR* and retinal biosynthesis gene cluster into the RpoS1 stress response regulatory network. This association appears to be unique at the present time as recent transcriptome analyses have revealed large photostimulons that do not include modulated *rpoS* expression in *pR*-containing *Dokdonia* strain MED134 [16] and *Candidatus* Pelagibacter ubique [29]. This is not entirely surprising as the acquisition of *pR* by different species via horizontal gene transfer likely leads to species-specific integration and regulation.

It is not known is whether light regulates *rpoS1* expression via activation of photoregulators or light sensing domains or whether it is indirectly responsible for its regulation as a photodamaging agent that induces the general cellular stress response. For example, blue light has been demonstrated to activate the general stress response in *Bacillus subtilis* via a LOV domain containing photoreceptor and the sigma^B^ transcription factor (a transcription factor that is also activated by entry into the stationary phase like RpoS1) [32]. However, the *V. campbellii* genome does not possess known photoactive domain (e.g. BLUF and LOV) containing proteins, photoactive yellow proteins or phytochromes [33]. Light has also been demonstrated to induce the sigma factor sigma^LitS^ that directs transcription of carotenoid biosynthesis in *Streptomyces coelicolor* A3(2) [34] and light-dependent photo-oxidative stress is a known inducer of sigma^E^ activity in *Rhodobacter sphaeroides* [35]. Given these findings and the suggestion that PRs could have a range of physiological functions [36], is it possible that the carotenogenesis required for PR holoprotein pigmentation seen in *V. campbellii* can also play a role in protecting against the deleterious effects of photo-oxidative stress by scavenging harmful reactive oxygen species?

In this study, we have demonstrated the expression, function, regulation and potential importance of *V. campbellii* BAA-1116 PR in a laboratory environment. Recent comparative metaproteomic analyses of microbial membrane fractions collected from South Atlantic surface waters have revealed the *in situ* expression of PR peptide sequences that were most closely related to *V. campbellii* BAA-1116 PR [37] suggesting the likely importance of *Vibrio* PRs in natural environments as well. An assessment of 11 other geographically, environmentally and temporally distributed *V. campbellii* isolates revealed that three [type strain ATCC 25920 (seawater isolate, Hawaii, USA), UWM E1 (a fish gut commensal, Chub Cay, Bahamas) and CAIM 1500 (fish liver isolate, Sinaloa, Mexico)], were also found to contain *pR* and retinal biosynthesis genes (manuscript in preparation). That 25% of the *V. campbellii* isolates tested have been shown to harbor the *pR* and retinal biosynthesis gene cluster indicates that this is not an unusual acquisition in this species. Overall, as a strategy for environmental adaptation, the importance of horizontal gene transfer in the evolution of several aspects of *Vibrio* biology, ranging from pathogenicity to multidrug resistance to niche specialization and now phototrophy, cannot be underestimated.

## Materials and Methods

### Proton-pumping measurements

*V. campbellii* WT and Δ*pR* strains were grown in Autoinducer Bioassay (AB) medium (ATCC medium 2034) for 18 hr in a 30°C shaking incubator (Materials and Methods S1). Prior to experimentation, cells were harvested via centrifugation, washed once in salt solution A (10 mM NaCl, 10 mM MgCl_2_ and 100 µM CaCl_2_, pH 7.0) and resuspended in salt solution A to a concentration of 1×10^10^ cells/ml. Five ml cell suspensions were placed in a 25 ml glass vial and measurements were taken immediately upon pH stabilization. The *V. campbellii* suspensions were illuminated with a Cuda I-150 quartz halogen fiberoptic lightsource (Sunoptic Technologies, Jacksonville, FL, USA). The illuminator was placed 4 cm from the surface of the vial with (480DF40, 530DF30, 530DF35, 670DF40, Omega Optical, Brattleboro, VT, USA) or without a bandpass filter. Continuous, real-time measurements of pH were done using an Oakton pH 510 series pH meter (Oakton Instruments, Vernon Hills, IL, USA). The output of the pH meter, a±2 V signal directly proportional to the measured pH, was fed into a LPF-8 differential amplifier with 8-pole low-pass Bessel filter (Warner Instruments, Hamdon, CT, USA). The output of the amplifier was then digitized using a Windows XP-based computer equipped with a DigiData 1322A data acquisition system and pClamp v9 software (Molecular Devices, Sunnyvale, CA, USA). Experiments were carried out in a benchtop Faraday cage to reduce electrical noise. The output signal of the pH meter was amplified using a gain of 100 and filtered at 20 Hz. Data was collected at a rate of 100 Hz, with the final results plotted at one second intervals as the mean of 100 data points. All light output measurements were taken with a Newport 815 Series optical digital power meter (Newport, Irvine, CA, USA).

### Quantative reverse transcription PCR

Real-time reverse transcription PCR assays were conducted on an iCycler (BioRad Laboratories, Hercules, CA, USA) using the OneStep RT-PCR kit (Qiagen, Valencia, CA, USA). Real-time PCR reaction mixtures consisted of 1× SYBR Green PCR Master Mix (Applied Biosystems, Foster City, CA, USA), 20 ng of total RNA from each extracted sample, 200 nM of primers PR-L2-F2 (5′-AGTCGCATCCGCTACAGTTT-3′) and PR-5end-R1 (5′-AGCAGTGGGACGGTAAGTAGC-3′) and were subjected to the following cycling conditions: one cycle at 50°C for 30 min and 95°C for 15 min, followed by 40 cycles of 94°C for 15 sec, 52°C for 30 sec and 72°C for 30 sec. The standard curve used for calculating transcript number was made from amplifying a ten-fold serial dilution of a *pR-* or *rpoS1-*containing plasmid.

### ATP measurements

*V. campbellii* WT and Δ*pR* cultures were grown for 24 hr in AB medium, pelleted, rinsed twice in PBS and 1×10^9^ cells were resuspended in 1 ml PBS and stored in the dark at room temperature for 90 min. Both sets of cells were then illuminated for 10 min using green light (530±15.0 nm, 20.0 mW). Post-illumination, the cells were immediately diluted to 2.5×10^7^ cells per well in PBS and tested using the BacTiter-Glo™ Microbial Cell Viability Assay (Promega, Madison, WI, USA) in a manner similar to that which has been previously described [14]. Cellular ATP concentrations were calculated based on an ATP standard curve (1 µM to 10 pM) that was simultaneously generated according to the manufacturer's specifications. The level of endogenous bioluminescence was equal in both strains, considered as background and appropriately subtracted.

### Viability assay

Overnight *V. campbellii* WT and D*pR* cultures grown in AB medium were pelleted, rinsed in artificial seawater (3% salinity–Instant Ocean Aquarium Systems, Mentor, OH, USA) and resuspended to 1×10^2^ cells/µl in a final volume of 50 µl of room temperature ASW or ASW + 300 mM sodium azide. Cell suspensions were either wrapped in aluminum foil (dark) or subjected to 20 mW of white light and incubated at room temperature for 2 hr. Serial dilutions were then spread on Luria Marine plates in quadruplicate to determine the number of viable colony forming units. This experiment was performed a total of four times.

### Minimal medium cultures and flow cytometry

*V. campbellii* WT and Δ*pR* 50 ml cultures were grown in M9 minimal medium (1.1% 5× M9 minimal media salts, 1.5% NaCl, 0.4% glucose, 2 mM MgSO_4_, 0.1 mM CaCl_2_, pH 7.8) in baffled 125 ml Erlenmeyer flasks with aeration (200 rpm) in a Precision Illuminated Incubator Model 818 set at 30°C with continuous uniform white light illumination at 42 µmol photons s^−1^ m^−2^ (photosynthetically active radiation). *V. campbellii* WT and Δ*pR* cultures were initially inoculated with 7202 cells ± 104/µL and 7372 cells ± 97/µL, respectively. At 6, 12, 18, 24, 30 and 36 hr post-inoculation, two 1 ml aliquots were removed and the cells were labeled with the SYTO-9 green-fluorescent nucleic acid stain. The SYTO-9 stained cell populations were then counted with an Accuri C6 flow cytometer (Ann Arbor, MI, USA) equipped with 488 and 640 nm lasers and standard filter setup. The SYTO-9 stained bacterial samples were gated and 50,000 events were collected for each sample. Each sample was measured three times. The bacterial concentration was calculated by dividing the number of events collected by the volume determined from the microsphere standard.

## Supporting Information

Figure S1 **Genetic locus, sequence and phylogenetic analysis of** ***V. campbellii*** **proteorhodopsin.** (a) Organization of the proteorhodopsin (*pR*) and retinal biosynthesis (*crtEIBY*, *blh*) genetic locus on *V. campbellii* BAA-1116 chromosome 1. Arrows indicate the transcriptional orientation of each annotated gene. (b) 271 AA sequence of *V. campbellii* PR. Overlined sequence–antibody epitope; black arrow–SignalP 3.0-predicted signal peptide cleavage site; gray boxes–TMHMM-predicted transmembrane helices (tmh); green asterisk–green light-tuning Leu residue; underlined residues–conserved AA residues critical for proton translocation. Brackets at the end of each row denote the *V. campbellii* PR AA position number. (c) Neighbor-joining tree showing the phylogenetic position of *V. campbellii* PR relative to representative environmental sequences. Evolutionary distance analysis of 159 positions was computed using the Poisson correction method and MEGA4 program. Bootstrap values of >50% from 1000 simulations are shown to the left of each branch point. Strains with a Leu or Gln residue at position 105 are listed in green or blue, respectively. Strains with a Met at position 105 are listed in black and served as the psychrophilic bacterial outgroup. The scale bar represents the number of AA substitutions per site.
(PDF)

Figure S2 ***V. campbellii*** **PR expression and function in** ***E. coli*** **.** (a) Pigmentation resulting from the heterologous expression of *V. campbellii* PR in *E. coli* cells in the presence of 10 µM all*-trans* retinal. (1) *E. coli* BL21 (empty plasmid control); (2) *E. coli* PR_Met1_; (3) *E. coli* PR_Leu20_. (b) Western blot analyses for PR expression from (1) *E. coli* BL21, (2) *E. coli* PR_Met1_ and (3) *E. coli* PR_Leu20_. Parallel blots were probed with an anti-His-tag monoclonal antibody or an anti-PR monospecific polyclonal antibody (targeting peptide PR_Leu20-Phe33_ – Fig. S1b). (c) Photoinduced proton pumping by *E. coli* PR_Met1_ cell suspensions. Changes in pH were monitored in 2 min intervals in the presence (white regions) and absence (gray regions) of white light (525 mW). Black line, *E. coli* PR_Met1_; blue line, *E. coli* BL21. (d) Absorption spectra of retinal-reconstituted cell membranes from (1) *E. coli* BL21 and (2) *E. coli* PR_Met1_ (λ_max_ 523 nm). (e) Spectrally-tuned proton pumping by *E. coli* PR_Met1_ cell suspensions. Changes in pH were monitored in 1 min intervals in the absence (gray regions) or presence of red light (670±20 nm, 10.5 mW, red regions) or green light (530±17.5 nm, 5.8 mW, green regions).
(PDF)

Materials and Methods S1 **Supporting Materials and Methods.**
(DOC)
