# Supplementary figures and images for: Function and Regulation of Vibrio campbellii Proteorhodopsin: Acquired Phototrophy in a Classical Organoheterotroph
Source: PLoS One. 2012 Jun 7;7(6):e38749. doi: 10.1371/journal.pone.0038749 (PMC3380642; doi:10.1371/journal.pone.0038749)

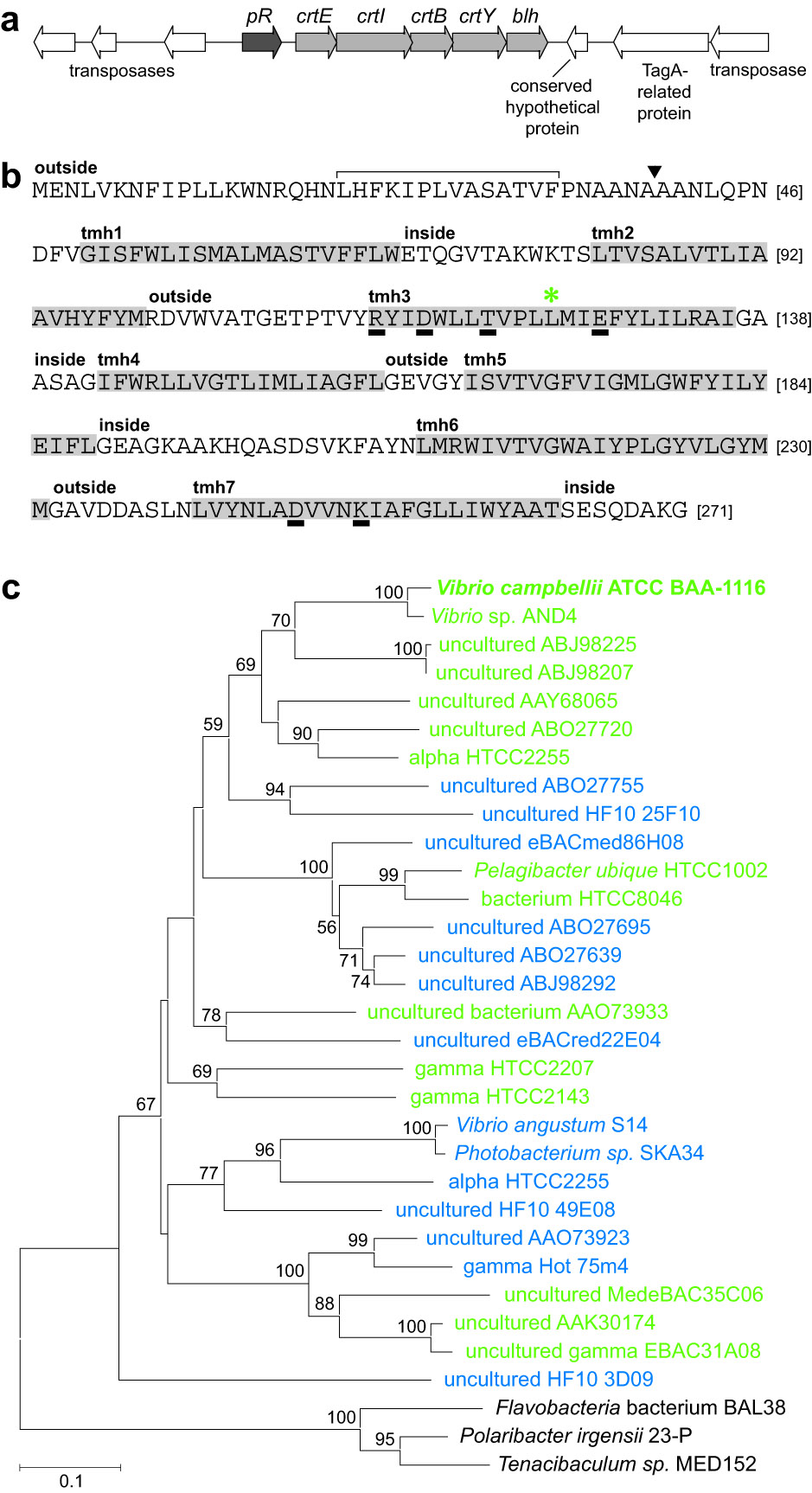

Supplement: Figure S1 — Genetic locus, sequence and phylogenetic analysis of V. campbellii proteorhodopsin. (a) Organization of the proteorhodopsin (pR) and retinal biosynthesis (crtEIBY, blh) genetic locus on V. campbellii BAA-1116 chromosome 1. Arrows indicate the transcriptional orientation of each annotated gene. (b) 271 AA sequence of V. campbellii PR. Overlined sequence–antibody epitope; black arrow–SignalP 3.0-predicted signal peptide cleavage site; gray boxes–TMHMM-predicted transmembrane helices (tmh); green asterisk–green light-tuning Leu residue; underlined residues–conserved AA residues critical for proton translocation. Brackets at the end of each row denote the V. campbellii PR AA position number. (c) Neighbor-joining tree showing the phylogenetic position of V. campbellii PR relative to representative environmental sequences. Evolutionary distance analysis of 159 positions was computed using the Poisson correction method and MEGA4 program. Bootstrap values of >50% from 1000 simulations are shown to the left of each branch point. Strains with a Leu or Gln residue at position 105 are listed in green or blue, respectively. Strains with a Met at position 105 are listed in black and served as the psychrophilic bacterial outgroup. The scale bar represents the number of AA substitutions per site. (PDF) [file pone.0038749.s001.pdf]

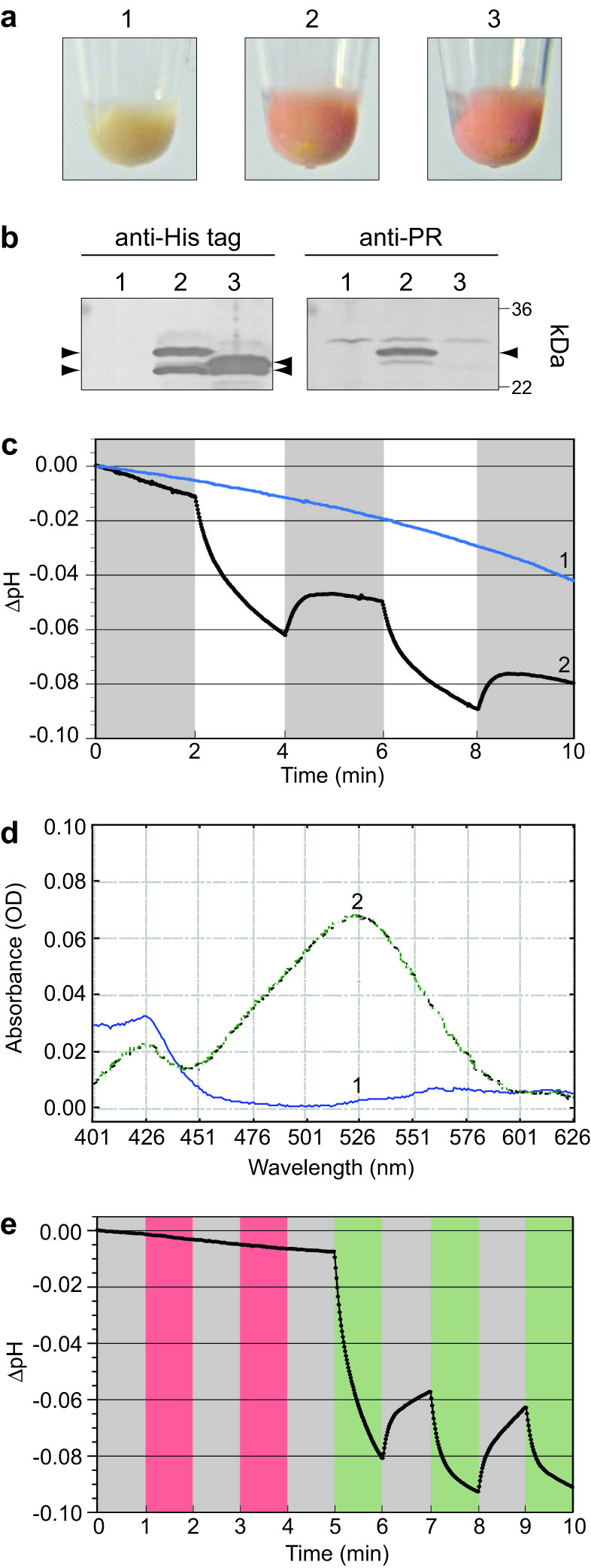

Supplement: Figure S2 — V. campbellii PR expression and function in E. coli . (a) Pigmentation resulting from the heterologous expression of V. campbellii PR in E. coli cells in the presence of 10 µM all-trans retinal. (1) E. coli BL21 (empty plasmid control); (2) E. coli PRMet1; (3) E. coli PRLeu20. (b) Western blot analyses for PR expression from (1) E. coli BL21, (2) E. coli PRMet1 and (3) E. coli PRLeu20. Parallel blots were probed with an anti-His-tag monoclonal antibody or an anti-PR monospecific polyclonal antibody (targeting peptide PRLeu20-Phe33 – Fig. S1b). (c) Photoinduced proton pumping by E. coli PRMet1 cell suspensions. Changes in pH were monitored in 2 min intervals in the presence (white regions) and absence (gray regions) of white light (525 mW). Black line, E. coli PRMet1; blue line, E. coli BL21. (d) Absorption spectra of retinal-reconstituted cell membranes from (1) E. coli BL21 and (2) E. coli PRMet1 (λmax 523 nm). (e) Spectrally-tuned proton pumping by E. coli PRMet1 cell suspensions. Changes in pH were monitored in 1 min intervals in the absence (gray regions) or presence of red light (670±20 nm, 10.5 mW, red regions) or green light (530±17.5 nm, 5.8 mW, green regions). (PDF) [file pone.0038749.s002.pdf]
