# Supplementary material for: Function and Regulation of Vibrio campbellii Proteorhodopsin: Acquired Phototrophy in a Classical Organoheterotroph
Source: PLoS One. 2012 Jun 7;7(6):e38749. doi: 10.1371/journal.pone.0038749 (PMC3380642; doi:10.1371/journal.pone.0038749)
Supplement: Materials and Methods S1 — Supporting Materials and Methods. (DOC) [file pone.0038749.s003.doc]

**Supplemental Materials and Methods**

*Construction of ΔpR deletion mutant*

The in-frame deletion of the *V. campbellii pR* gene was generated by overlap PCR (Warrens AN et. al. 1997. *Gene* 186(1):29-35) and the resulting 1 Kb PCR fragment was cloned into the pCR4-TOPO vector using the TOPO TA cloning kit (Invitrogen). This deletion fragment was digested from the TOPO vector with *Bam*HI and *Xho*I and cloned into the plasmid pZW125, which was constructed by inserting a chloramphenicol resistance gene into the oriR_R6Kg_ plasmid pWM91 containing the *sacB* gene (Metcalf WW*, et al.* 1996. *Plasmid* 35(1):1-13). The resulting plasmid (pZW024) was transformed into *E. coli* strain Sm10λpir and transferred into a *V. campbellii* spontaneous streptomycin resistant mutant (*V. campbellii*-str1) by conjugation. The conjugants were grown on Luria Marine (LM) agar plates containing 3 μg/ml chloramphenicol and 1 μg/ml streptomycin. The *pR* deletion mutant (Δ*pR*) was selected on LM plates supplemented with 6% sucrose and verified by PCR.

*Heterologous expression of V. campbellii PR and BPR*

The *pR* gene was amplified using primers PR_Met1_-F (5’-CACCATGGAAAATCTGGTTAAAAATTT-3’) / PR_Met1_-R (5’-TCCCTTGGCGTCCTGACTTT-3’) or PR_Leu20_-F (5’-CACCATGCACTTCAAGATACCACTAG-3’) / PR_Leu20_-R (5’-TCCCTTGGCGTCCTGACTTT -3’) and cloned into a pET101 TOPO expression vector (Invitrogen, Carlsbad, CA, USA). The resulting plasmids (pZW022 and pZW023, respectively) were transformed into *E. coli* BL21 (DE3) for *in vivo* expression. Both constructs were grown in LB medium supplemented with 100 μg/ml ampicillin in a 30°C shaking incubator and *pR* expression was induced in mid-log phase with 5 mM IPTG in the presence of 10 μM ethanolic all-*trans* retinal. Absorption spectra of induced cells and isolated cell membranes in phosphate buffered saline (pH 7.2) were recorded with a Cary 5000 UV-Vis Near IR spectrophotometer and data analysis was performed using the Cary Win UV Scan Application software version 3.00 (Varian, Palo Alto, CA, USA). Data ranging between 400-650 nm were smoothed and baseline corrected using Peak Fit software version 4.11.

*Immunoblot analyses*

Seventy-five μg of *E. coli* crude cell lysate proteins were electrophoresed through an 8-16% gradient SDS-PAGE gel (Invitrogen) and transferred to a PVDF membrane. Membranes were probed with a 1:2000 dilution of Anti-His (C-term)/AP antibody (Invitrogen) or a 1:2000 dilution of anti-*V. campbellii* PR_Leu20-Phe33_ peptide rabbit serum (GenScript Corp.) and developed using the WesternBreeze Chromogenic Immunodetection System (Invitrogen).

*Proton-pumping measurements*

The *E. coli* PR_Met1_ and BPR_PalE6_ constructs were grown in LB medium supplemented with 100 μg/ml ampicillin in a 30°C shaking incubator. In mid-log phase, only the *E. coli* cells were provided with 10 μM ethanolic all-*trans* retinal and PR expression was induced with 5 mM IPTG. The *E. coli* cells were prepared for experimentation 4 to 6 hr post-induction. Prior to experimentation, bacterial cells were harvested via centrifugation, washed once in salt solution A (10 mM NaCl, 10 mM MgCl_2_ and 100 µM CaCl_2_, pH 7.0) and resuspended in salt solution A to a concentration of 1x10^9^ cells/ml. Five ml cell suspensions were placed in a 25 ml glass vial and measurements were taken immediately upon pH stabilization. The *E. coli* suspensions were illuminated with a Fiber-Lite 180 fiber optic lamp (Dolan-Jenner Industries, Boxborough, MA, USA). The illuminator was placed 4 cm from the surface of the vial with (480DF40, 530DF30, 530DF35, 670DF40, Omega Optical, Brattleboro, VT, USA) or without a bandpass filter. For experiments where *V. campbellii* bioluminescent cultures served as the light source, the *V. campbellii* were placed into a 4.5" tall cylindrical chamber constructed around a 4" round aquarium airstone (Top Fin, Phoenix, AZ, USA). The *V. campbellii* PR or BPR-containing *E. coli* cultures to be tested were placed in a glass tube with a pH probe inserted and then suspended but submerged into the *V. campbellii* culture. Air was bubbled through the air stone in order to increase the oxygen content of the *V. campbellii* media to induce bioluminescence. Sessation of air flow resulted in the *V. campbellii* culture dimming over a period of a few minutes. Continuous, real-time measurements of pH were done using an Oakton pH 510 series pH meter (Oakton Instruments, Vernon Hills, IL, USA). The output of the pH meter, a ±2V signal directly proportional to the measured pH, was fed into a LPF-8 differential amplifier with 8-pole low-pass Bessel filter (Warner Instruments, Hamdon, CT, USA). The output of the amplifier was then digitized using a Windows XP-based computer equipped with a DigiData 1322A data acquisition system and pClamp v9 software (Molecular Devices, Sunnyvale, CA, USA). Experiments were carried out in a benchtop Faraday cage to reduce electrical noise. The output signal of the pH meter was amplified using a gain of 100 and filtered at 20 Hz. Data was collected at a rate of 100 Hz, with the final results plotted at one second intervals as the mean of 100 data points. All light output measurements were taken with a Newport 815 Series optical digital power meter (Newport, Irvine, CA, USA).

*V. campbellii membrane fraction preparation and mass spectrometry analysis*

*V. campbellii* cell pellets were resuspended in 100 mM Na_2_CO_3_, pH 11.4 with Roche Complete Protease Inhibitor Cocktail Tablets and sonicated for 1 min using a Branson Sonifier at setting 7. The cell suspension was allowed to cool for 2 min on ice and this cycle was repeated four more times. The cell lysate was then centrifuged at 4000 x *g* for 15 min and then diluted to 30 ml with ice-cold 100 mM Na_2_CO_3_, pH 11.4 and incubated for 1 hr at 4ºC with agitation and the cell membranes were subsequently pelleted via ultracentrifugation at 100,000 x *g* for 1 hr. Membrane pellets were resuspended in 2% SDS using minimal sonication and the protein concentration was determined using the DC Protein Assay (Bio-Rad). Forty µg of protein were loaded per well, electrophoresed through a Novex 8-16% gradient SDS-PAGE gel (Invitrogen) and visualized using coomassie blue staining. Protein bands from the SDS-PAGE experiments were excised and subjected to a standard in-gel tryptic digest protocol (Jensen ON et al. 1999. *Methods Mol Biol* 112:513-530). Following peptide extraction, the samples were evaporated to dryness and desalted using a reverse phase SPE TopTip (Glygen, Columbia, MD, USA) and resuspended in reverse phase mobile phase A (97.5% H2O, 2.4% ACN, 0,1% formic acid) in preparation for LC-MS. LC-MS was performed using an LC Packings Ultimate nanoLC system coupled to an Applied Biosystems Qstar Pulsar I and standard reverse phase conditions. The integrated software Analyst 1.1 was used for the acquisition of the mass spectrometry data using the IDA data dependent mode. The Applied Biosystems ProteinPilot™ software v. 2.0 was used to interrogate all MS/MS data. The raw MS and MS/MS data were searched against an in-house database constructed using the translated genomic sequence of *V. campbellii* BAA-1116. Protein identifications with confidences of ≥ 99% were used to confirm identification.
